# Osthole Attenuates Doxorubicin-Induced Apoptosis in PC12 Cells through Inhibition of Mitochondrial Dysfunction and ROS Production

**DOI:** 10.1155/2014/156848

**Published:** 2014-06-12

**Authors:** Yalda Shokoohinia, Leila Hosseinzadeh, Maryam Moieni-Arya, Ali Mostafaie, Hamid-Reza Mohammadi-Motlagh

**Affiliations:** ^1^Novel Drug Delivery Research Center, School of Pharmacy, Kermanshah University of Medical Sciences, Kermanshah 6734667149, Iran; ^2^Department of Pharmacognosy and Biotechnology, School of Pharmacy, Kermanshah University of Medical Sciences, Kermanshah 6734667149, Iran; ^3^Students Research Committee, School of Pharmacy, Kermanshah University of Medical Sciences, Kermanshah 6734667149, Iran; ^4^Medical Biology Research Center, Kermanshah University of Medical Sciences, Kermanshah 6714869914, Iran

## Abstract

Doxorubicin (DOX) is a potent, broad-spectrum chemotherapeutic drug used for treatment of several types of cancers. Despite its effectiveness, it has a wide range of toxic side effects, many of which most likely result from its inherent prooxidant activity. It has been reported that DOX has toxic effects on normal tissues, including brain tissue. In the current study, we investigated the protective effect of osthole isolated from *Prangos ferulacea* (L.) Lindl. on oxidative stress and apoptosis induced by DOX in PC12 as a neuronal model cell line. PC12 cells were pretreated with osthole 2 h after treatment with different concentrations of DOX. 24 h later, the cell viability, mitochondrial membrane potential (MMP), the activity of caspase-3, the expression ratio of Bax/Bcl-2, and the generation of intracellular ROS were detected. We found that pretreatment with osthole on PC12 cells significantly reduced the loss of cell viability, the activity of caspase-3, the increase in Bax/Bcl-2 ratio, and the generation of intracellular ROS induced by DOX. Moreover, pretreatment with osthole led to an increase in MMP in PC12 cells. In conclusion, our results indicated that pretreatment with nontoxic concentrations of osthole protected PC12 cells from DOX-mediated apoptosis by inhibition of ROS production.

## 1. Introduction

Doxorubicin (DOX), an antibiotic produced by the fungus* Streptomyces peucetius*, is a potent anticancer drug commonly used in the treatment of a variety of cancers [1]. In addition to its potent antitumor activity, DOX is associated with a number of unwanted side effects on nonspecific organs such as the heart and brain [2–4]. Despite the well-known side effects of DOX treatment related to the heart, little is known about its effects on the brain. Although the distribution of doxorubicin into the brain is low, its penetration can increase when the blood-brain barrier is temporarily disrupted following administration of mannitol, morphine, dexamethasone, or ondansetron. In the brain, the toxicity of doxorubicin diffusely occurs with injury of the neurons in the cortex and subcortical nuclei of the brain [5]. The mechanisms by which doxorubicin induces neuronal cell death are unclear; however, some evidence indicates that DOX toxicity in neural cells follows the generation of free radicals [6]. Compared with other body tissues, neural cells are highly sensitive to oxidative damage because of their poor antioxidant defenses [7].

Coumarins are heterocyclic phenolics that have been associated with beneficial effects on human health, such as reducing the risk of cancer, diabetes, cardiovascular diseases, and brain diseases because of their radical scavenging and antioxidant activities [8–10].

Osthole (7-methoxy-8-isopentenoxy-coumarin), which is extracted from* Prangos ferulacea *(L.) Lindl., has been widely reported to have pharmacological activities such as antispasmodic [11] anticonvulsant [12], and blood pressure and lipid-reducing effects [13]. Some researchers have reported that osthole had a neuroprotective effect against traumatic brain injury and accumulation of *β*-amyloid peptide injuries [14, 15]. In addition, a previously published study indicated that osthole increased DOX-induced apoptosis in human breast carcinoma cells [16]. No data are available on whether these effects are related to tumor cells or can be observed in normal neuronal cells. Therefore, the current study was designed to investigate the effects of osthole on DOX-induced cytotoxicity in PC12 cells, as a widely accepted model of neuronal cells [17, 18].

## 2. Material and Methods

### 2.1. Materials

Fluorescent probe 2,7-dichlorofluorescein diacetate (DCF-DA), 3-(4,5-dimethylthiazol-2-yl)-2,5-diphenyl tetrazolium (MTT), triton X-100, FBS, and rhodamine 123 were purchased from Sigma (St Louis, MO, USA). DMEM-F12 was purchased from Gibco (Gibco, Grand Island, NY, USA). Caspase-3 Detection Kit was provided from Sigma. Express One-Step SYBR GreenER Kit was purchased from Invitrogen (Carlsbad, CA).

### 2.2. Plant Material and Isolation of Osthole

**Figure 1:**
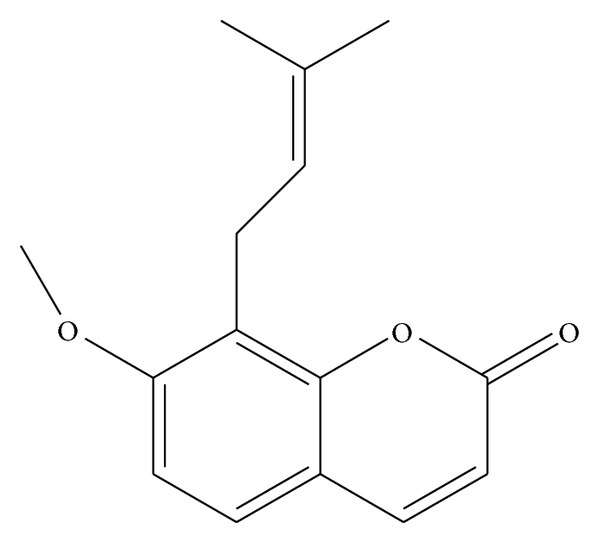
Chemical structure of osthole.

Osthole was isolated from* Prangos ferulacea *(L.) Lindl. roots. Plant material gathering, identification and extraction, and isolation of osthole (Figure 1) were performed as previously reported [19]. Structure of the compound was elucidated by using ^1^H-NMR, ^13^C-NMR, and Mass spectra and comparing to literature [20, 21].

### 2.3. Cell Culture

Rat pheochromocytoma-derived cell line PC-12 was obtained from Pasteur Institute (Tehran, Iran). The PC12 cell line is widely used as a model for dopaminergic neurons because it possesses intracellular substrates for the synthesis, metabolism, and transportation of dopamine (DA). This makes PC12 cells useful as a model system for neuroprotection study [22]. The cells were maintained at 37°C in 95% CO_2_ humidified incubator. Cells were cultured in Dulbecco's modified Eagle's medium (DMEM) with 10% (v/v) heat-inactivated fetal bovine serum, 100 UmL^−1^ penicillin, and 100 mg/mL^−1^ streptomycin.

### 2.4. Cell Viability

Cellular toxicities of DOX and osthole were analyzed in PC-12 cells using the methylthiazolyltetrazolium bromide (MTT) method. Cells were cultured in a 96-well microplate at a density of 7,000 cells/well and in a volume of 200 *μ*L. Stock solutions of DOX and osthole were prepared in dimethyl sulfoxide (DMSO). At appropriated time intervals, the medium was removed and replaced by 200 *μ*L of 0.5 mg/mL of MTT in growth medium and then the plates transferred to a 37°C incubator for 2–4 h. Then, the medium was removed, and the purple formazan crystals were dissolved in DMSO (200 *μ*L/well). Absorbance was determined on an ELISA plate reader (BioTek, H1M) with a test wavelength of 570 nm and a reference wavelength of 630 nm to obtain sample signal (OD570–OD630). Also, the morphological changes of the cells were observed using phase contrast inverted microscope (Motic, China) at 40x magnifications.

### 2.5. Measurement of Mitochondrial Membrane Potential

Mitochondrial dysfunction has been shown to participate in the induction of apoptosis. In this study, mitochondrial membrane potential (MMP) was measured by using rhodamine 123 fluorescent dye. Depolarization of MMP during cell apoptosis results in the loss of rhodamine 123 from the mitochondria and a decrease in intracellular fluorescence intensity [23]. Cells were incubated with rhodamine 123 for 30 min at 37°C. The fluorescence was measured at an excitation wavelength of 488 nm and an emission wavelength of 520 nm using a fluorescence microplate reader (BioTek, H1M, USA).

### 2.6. Real-Time RT-PCR Analysis of Apoptosis-Related Gene Expression

Total RNA from PC12 cells were extracted using high pure isolation kit (Roche, Mannheim, Germany) according to the manufacturer's instructions. Quality and quantity of total RNA were assessed by spectrophotometer (NanoDrop 2000, USA) and samples were stored at −80°C until use. The primers used in this study were selected from published studies [1]. The performances of all primer pairs were tested by primer concentration to determine the optimal reaction conditions. Thermal cycler conditions were 15 min at 50°C for cDNA synthesis, 10 min at 95°C followed by 40 cycles of 15 s at 95°C to denature the DNA, and 45 s at 60°C to anneal and extend the template. Melting curve analysis was performed to ascertain specificity by continuous acquisition from 65°C–95°C with a temperature transient rate of 0.1°C/S. All reactions were performed in triplicate in a Corbett system (Australia). The values obtained for the target gene expression were normalized to *β*-actin and analyzed by the relative gene expression −ΔΔCT method where *‒*ΔΔCT = (CT target − CT *β*-actin) Unknown − (CT target − CT *β*-actin) Calibrator.

### 2.7. Assay for Caspase-3 Activity

Caspase-3 assay was carried out using the Sigma colorimetric caspase kit. This assay was based on the ability of the active enzyme to cleave the chromophore from the enzyme substrate, Ac-DEVD-pNA in equal amount of cells protein. The cells (5 × 10^6^) were harvested and lysed in 70 *μ*L of the cell lysis buffer included with the kit, and protein concentrations were equalized for each condition. Subsequently, 10 *μ*L of cell lysate was combined with an equal amount of substrate reaction buffer containing a caspase-3 colorimetric substrate. This mixture was incubated for 2 h at 37°C, and then the pNA light emission was quantified using a microplate reader at 400 or 405 nm (BioTek, H1M.).

Comparison of the absorbance of pNA from an apoptotic sample with an uninduced control allowed determination of the fold increase in caspase-3 activity. The protein content was determined by the Bradford method using the bovine serum albumin as a standard.

### 2.8. Determination of Intracellular ROS

Intracellular ROS levels were examined using DCF-DA. DCF-DA is a nonfluorescent lipophilic ester that easily crosses the plasma membrane. Into the cytosol, the acetate group is rapidly removed by unspecific esterases. The oxidation of this molecule to the fluorochrome DCF results in green fluorescence. The intensity of this fluorescence is generally considered to reflect the level to which ROS are present [24].

Briefly, after seeding for 24 h, PC12 cells were washed with PBS buffer (pH 7.4). The cells pretreated with osthole were then treated with DOX for an additional 24 h. After washing with PBS, the cells were incubated with 20 *μ*L DCF-DA at 37°C for 30 min. After incubation, cells were lysed with Triton X-100. The fluorescence was measured at an excitation wavelength of 488 nm and an emission wavelength of 535 nm using a fluorescence microplate reader (BioTek, H1M, USA).

### 2.9. Statistical Analysis

Each experiment was performed at least three times, and the results were presented as mean ± S.E.M. One-way analysis of variance (ANOVA) followed by Tukey's test was used to compare the differences between means. A probability value of *P* < 0.05 was considered to be statistically significant.

## 3. Results

### 3.1. Cell Viability after Exposure to DOX and Coumarins Alone

**Figure 2:**
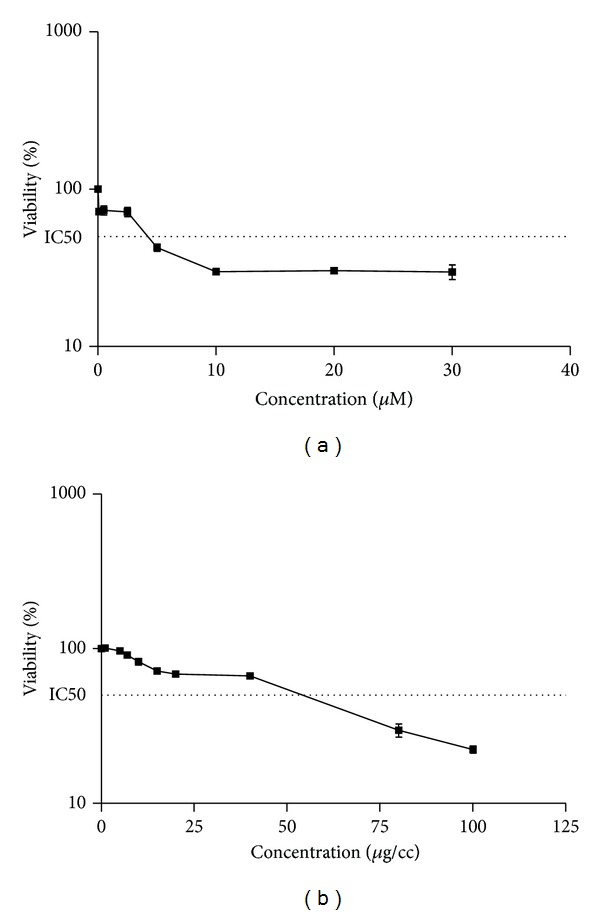
The effect of (a) DOX and (b) osthole on PC12 cells viability. The cell viability was determined by MTT assay as described in Section 2. Data are expressed as the mean ± S.E.M of three separate experiments.

The viability of cells was evaluated after 24 h of exposure to different concentrations of DOX. As shown in Figure 1 DOX induced cytotoxicity in a concentration dependent manner (Figure 2(a)). In order to set coumarins at concentrations which are nontoxic to cells but could prevent DOX-induced cytotoxicity, we also examined the effects of different concentrations of osthole on cell viability on PC12 cells. The percentage of cell viability has been shown in Figure 2(b). The figure clearly revealed that exposure to osthole induced cytotoxicity at the concentrations more than 15 *μ*g/cc.

### 3.2. Effect of Osthole Pretreatment on DOX-Induced Cell Death

**Figure 3:**
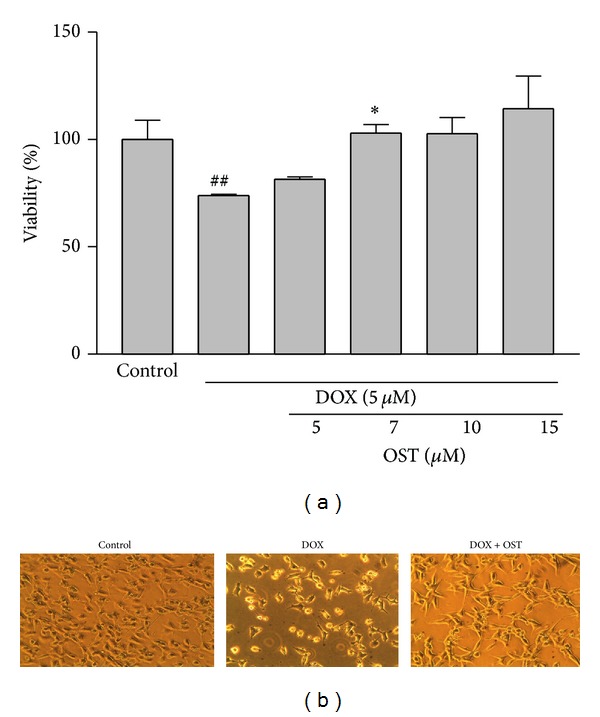
The effect of osthole on DOX-induced cytotoxicity in PC12 cells. (a) Cells were pretreated with different concentrations of osthole 2 h before exposure to 5 *μ*M of DOX. Data are expressed as the mean ± S.E.M of three separate experiments. ^##^ *P* < 0.01 versus control, *P < 0.05 versus DOX-treated cells. (b) Representative photomicrograph shows morphological changes of cells. Cells were pretreated with osthole (7 *μ*g/cc) for 2 h before exposure to 5 *μ*M of DOX and imaged by inverted phase contrast microscope.

For evaluation of effect of osthole pretreatment on DOX-induced cytotoxicity, PC12 cells were pretreated with different concentrations of osthole (5–15 *μ*g/cc) for 2 h; then the medium was changed and cells were treated with IC50 concentration of doxorubicin (5 *μ*M) for another 24 h. As shown in Figure 3(a), compared to control, the presence of osthole alleviates the extent of cell death which was significant at the 7 *μ*g/cc concentrations of the coumarin. Based on these results, pretreatment with 7 *μ*g/cc of osthole was selected for further experiments. Moreover, morphological assessment by inverted microscope showed that, after 24 h of exposure with DOX (5 *μ*M), cell population was greatly decreased, moderate cytoplasmic granulations were noticeable, and a large number of cells became rounded and started to detach from the flasks. Compared with the group treated with DOX alone, the cell population in the groups that had been pretreated with osthole (7 *μ*g/cc) increased (Figure 3(b)).

### 3.3. Effects of Osthole on Mitochondrial Membrane Depolarization (MMP) Induced by DOX

**Figure 4:**
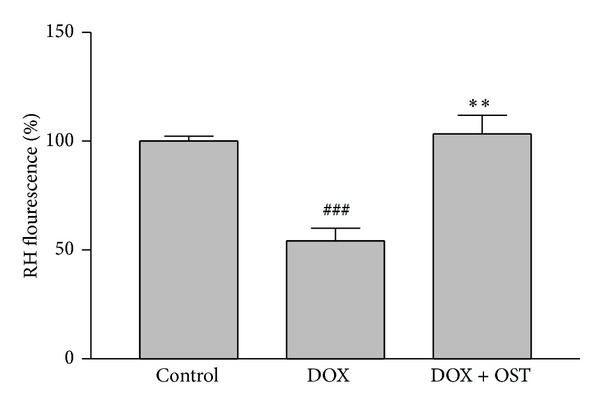
Effect of osthole on DOX-induced mitochondrial membrane potential (MMP) collapse. Cells were pretreated with osthole (7 *μ*g/cc) 2 h before exposure to 5 *μ*M of DOX. Graphs showing the change in MMP as represented by the mean fluorescence intensity (MFI) of rhodamine 123. ^###^ *P* < 0.001 versus control group. **P < 0.01 versus group treated with DOX alone.

Mitochondrial membrane depolarization (MMP) was determined using a cell permeable cationic fluorescent dye [23]. Depolarization of mitochondria membrane potential induced by the DOX-induced damage of the outer membrane resulted in the loss of the dye from the mitochondria and a decrease in intracellular fluorescence compared with the group treated with DOX alone (54.22 ± 4.8%); fluorescent intensities increased to 109.6 ± 9.1% after the use of 7 *μ*g osthole pretreatment (Figure 4).

### 3.4. Effects of Osthole Pretreatment on mRNA Expression of Bax and Bcl-2

To investigate how osthole pretreatment decreases DOX-induced apoptosis, we examined the mRNA expression of Bcl-2 protein family (Bcl2 and Bax) in PC12 cells.

**Figure 5:**
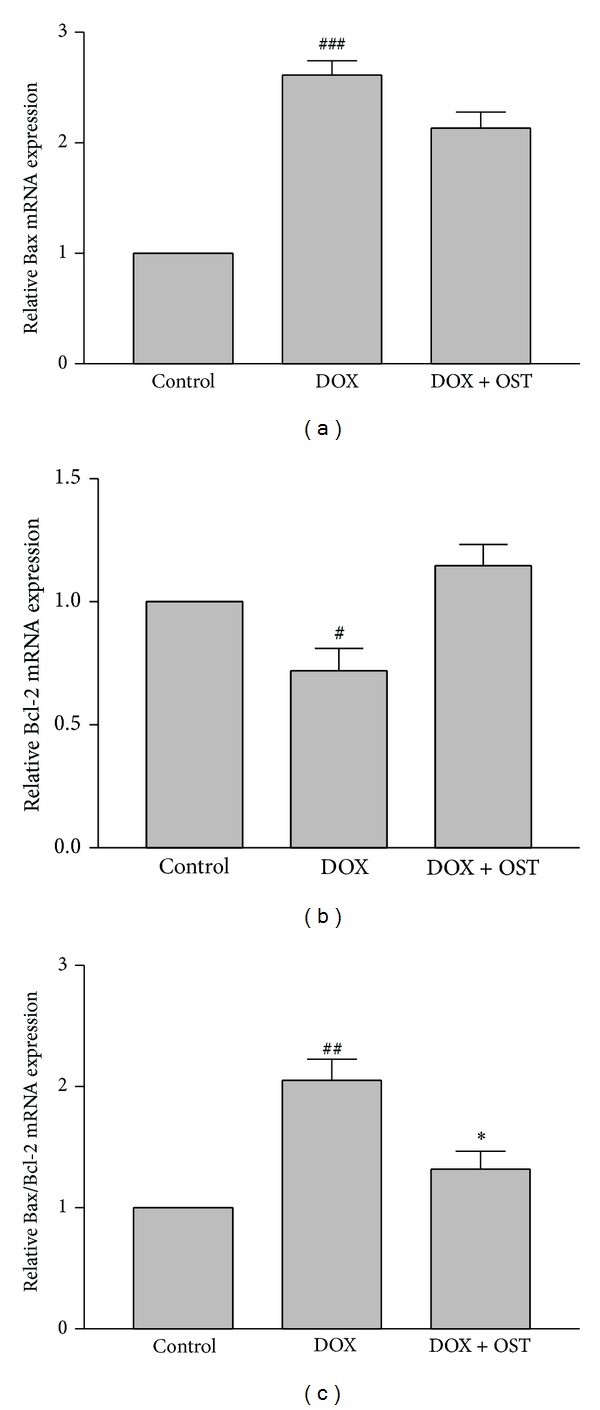
The effect of osthole on (a) Bax, (b) Bcl-2 mRNA expression, and (c) Bax/Bcl-2 in PC12 cells. Cells were pretreated with osthole (7 *μ*g/cc) 2 h before exposure to 5 *μ*M of DOX. Normalization relative to B-actin was performed. Levels of mRNA are expressed relative to control cells in the mean ± S.E.M values derived from three independent experiments. ^#^ *P* < 0.05, ^##^ *P* < 0 < 0.01, ^###^ *P* < 0.001 versus control, and *P < 0.05 versus DOX-treated cells.

Real-time RT-PCR analysis clearly shows a significant reduction of the expression level of Bcl-2 after treatment with DOX. Moreover, induction of apoptosis by DOX was accompanied with increase in proapoptotic Bax level. When we examined the effect of osthole pretreatment, we found that pretreatment of PC12 cells with osthole had no effect on mRNA expression of Bax and Bcl-2. The Bax/Bcl-2 ratio is a better apoptotic index than the two proteins considered separately. Our results showed that the Bax/Bcl2 ratio increased 2.31-fold upon treatment with doxorubicin, while, in cells that had been pretreated with osthole, the Bax/Bcl2 ratio markedly decreased to 1.18 (Figure 5).

### 3.5. Effect of Osthole Pretreatment on Caspase-3 Activation

**Figure 6:**
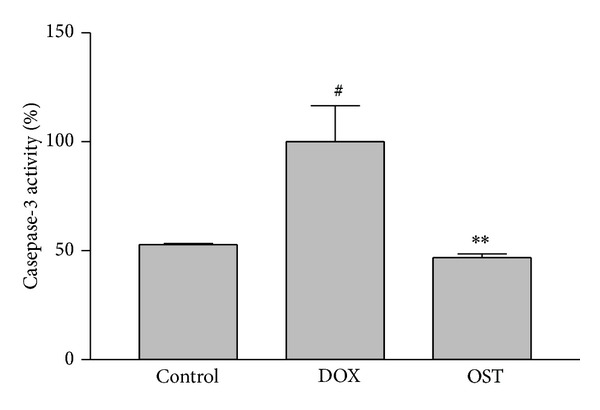
The effect of osthole on caspase-3 activity in PC12 cells. Cell pretreated with osthole 2 h before exposure to 5 *μ*M of DOX. Caspase-3 activity was measured by colorimetric detection of p-nitroanilide and was expressed as percentage of control. Data are expressed as mean ± S.E.M of three separate experiments. ^###^ *P* < 0.001 versus control and ***P < 0.001 versus DOX-treated cells.

Activation of caspase cascade is critical in the initiation of apoptosis in various biological systems [25]. A member of this family caspase-3 has been identified as being a key mediator of apoptosis in mammalian [25]. The obtained results showed that DOX significantly increased caspase-3 activation in PC12 cells. Pretreatment with osthole decreased caspase-3 activation significantly compared to DOX-treated cells (Figure 6).

### 3.6. Effects of Osthole on Oxidative Stress Induced by DOX

**Figure 7:**
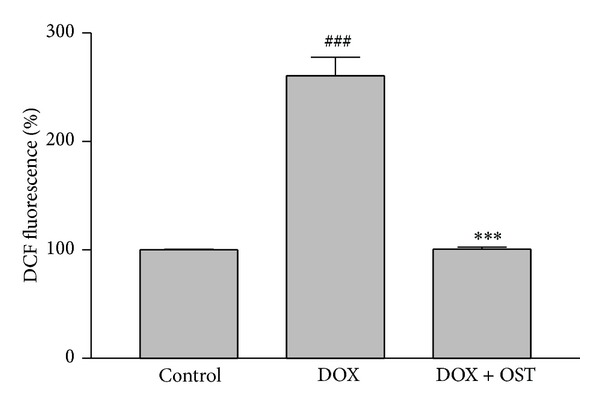
Effect of osthole on DOX-induced ROS overproduction. Cell pretreated with osthole 2 h before exposure to 5 *μ*M of DOX. The fluorescence intensity of DCF was measured in a microplate reader at an excitation wavelength of 488 nm and an emission wavelength of 535 nm. The data were represented as mean ± S.E.M for three independent experiments. *P < 0.05 and **P < 0.01 versus control group. ^#^ *P* < 0.05 and ^##^ *P* < 0.01 versus group treated with DOX alone.

In order to measure oxidative stress induced by DOX, fluorescent dye DCF-DA was used to measure ROS production. As anticipated, treatment of PC12 cells with DOX significantly increased ROS after 24 h of exposure in comparison to control. We investigated the inhibitory effect of osthole on ROS production by DOX. As it can be observed in Figure 7, osthole significantly decreased the DOX-induced formation of ROS.

## 4. Discussion

Natural products have played a significant role not only in the clinical nutrition against several diseases but also in drug discovery and development, contributing to finding alternative therapies [26]. In the current study, we evaluated the neuroprotective effect of osthole isolated from* P. ferulacea* on DOX-induced oxidative stress and apoptosis in PC12 cells.

Results showed that DOX decreases cell viability in PC12 cells. Furthermore, our results confirmed that DOX-mediated cytotoxicity is mainly affected by apoptosis. The Bcl-2 family of proteins is a key regulatory component of the cell death process, acting to either inhibit or promote cell death [1]. Bax and Bcl-2, the two main members of this family, influence the permeability of mitochondrial membranes. Cell survival in the early phases of the apoptotic cascade depends mostly on the balance between the proapoptotic and antiapoptotic proteins of the Bcl-2 family [27]. In the current study, DOX induced apoptosis by upregulating the proapoptotic gene* Bax*. The Bax/Bcl-2 ratio is an important regulator of apoptosis by decreasing the mitochondrial membrane potential. Permeabilization of the mitochondrial membrane causes bioenergetic catastrophe and permits the release of soluble molecules from the outer space of the mitochondria to the cytosol, which in turn leads to the activation of caspases, a family of cysteine proteases [28]. Caspase-3 is the ultimate executioner caspase that is necessary for the nuclear changes related to apoptosis [29].

In the current study, DOX significantly increased caspase-3 activation compared with controls. This is in line with an earlier study that showed that DOX can induce neural cell death by apoptosis in primary neural culture [3]. When we examined the protective effect of osthole on the cytotoxicity induced by DOX in PC12 cells, we observed that pretreatment of PC12 cells with a subtoxic concentration of this compound markedly protected the cells from DOX-induced cytotoxicity. Pretreatment with osthole reduced the DOX-induced Bax/Bcl-2 ratio and led to an increase in the mitochondrial membrane potential in PC12 cells. Because of the important role of mitochondria in the apoptotic cascade, it is not surprising that osthole pretreatment is also associated with inhibition of downstream apoptotic signaling pathways and ultimately suppresses the activation of caspase-3.

Reactive oxygen species have been implicated in the pathogenesis of several types of neurodegenerative disorders. Moreover, free radical-mediated oxidative stress has been proposed as a potential mechanism underlying DOX-induced apoptosis in the brain [5]. Previous studies show that the DOX treatment promotes lipid peroxidation in brain, heart, liver, lung, and kidney tissues [1, 30]. Cardoso et al. have previously reported that DOX treatment increased the formation of TBARS in brain mitochondria isolated from DOX-treated rats [4]. Furthermore, Öz and Ilhan [31] reported that DOX-treated rats undergoing cotreatment with melatonin, a free radical scavenger and antioxidant, presented lower levels of lipid peroxidation in kidney, lung, liver, and brain tissues compared with those in controls. In accordance with these findings, evidence from the current investigation suggests that ROS plays an important role in DOX-induced apoptosis in this model. Therefore, we decided to evaluate ROS production after pretreatment with osthole. The results indicated that the neuroprotective effect of osthole was mediated by the reduction of ROS levels. The present findings corroborate similar findings by Liu et al. who reported that osthole ameliorates MPP^+^-induced apoptosis in PC12 cells via inhibition of ROS [15]. In addition, another study has shown that osthole exhibits therapeutic potential for vascular dementia, which is most likely related to its antioxidation and antiapoptotic actions [32].

In summary, our results suggest that osthole attenuates the oxidative stress injury and apoptosis induced by DOX in PC12 cells. However, further studies are necessary to determine the exact neuroprotective mechanisms before definite conclusions can be drawn.
